# OMNIS: a spatially informed multi-omics deep-learning framework for tumor recurrence prediction and primary–metastatic tumor differentiation title page

**DOI:** 10.3389/frai.2026.1817043

**Published:** 2026-07-15

**Authors:** Junxian Li, Yuchen Xing, Ximin Gao, Renhe Liu

**Affiliations:** 1 Department of Blood Transfusion, Key Laboratory of Cancer Prevention and Therapy, Tianjin, Tianjin’s Clinical Research Center for Cancer, Tianjin Medical University Cancer Institute & Hospital, National Clinical Research Center for Cancer, Tianjin Medical University, Tianjin, China; 2 Department of Cancer Epidemiology and Biostatistics, Key Laboratory of Molecular Cancer Epidemiology, Tianjin, Tianjin’s Clinical Research Center for Cancer, Tianjin Medical University Cancer Institute & Hospital, National Clinical Research Center for Cancer, Tianjin Medical University, Tianjin, China; 3 Department of Diagnostic and Therapeutic Ultrasonography, Key Laboratory of Cancer Prevention and Therapy, Tianjin, Tianjin’s Clinical Research Center for Cancer, Tianjin Medical University Cancer Institute & Hospital, National Clinical Research Center for Cancer, Tianjin Medical University, Tianjin, China

**Keywords:** cancer recurrence prediction, convolutional deep learning, metastasis classification, multi-omics integration, three-dimensional genome organization

## Abstract

**Background:**

Cancer recurrence and distant metastasis are major causes of cancer-related death, yet existing biomarkers and single-omics models have limited accuracy and interpretability across tumor types.

**Methods:**

We developed OMNIS (OMics Network Integration and Spatial representation), a convolutional deep-learning framework that embeds multi-omics profiles into a five-channel genomic image ordered by Hi-C–derived chromosomal proximity. Somatic mutation, copy-number alteration, DNA methylation and gene-expression data from 1,578 TCGA tumors across 33 cancer types were used to train classifiers for recurrence risk and for primary-versus-metastatic status. Performance was assessed by 10-fold cross-validation using AUROC, AUPR and threshold-based metrics. Integrated gradients yielded per-gene attribution scores; top-ranked genes were evaluated for prognostic value in two independent non-small cell lung cancer cohorts (GSE31210, *n* = 226; GSE135222, *n* = 27) using survival analyses.

**Results:**

OMNIS achieved high discrimination for recurrence (AUROC/AUPR 0.970/0.937) and metastasis (0.980/0.883), with accuracies of 0.873–0.911 and negative predictive values ≥0.970 across tasks. Spatial genomic embedding accelerated convergence and outperformed non-spatial baselines. Attribution highlighted seven recurrence-associated genes (including IBA57, DNTTIP1, SLC20A2 and TMEM201) and ten metastasis-associated genes (including PLXNA1, POLR3D, TTLL4, SREBF2, TYMP and ZBTB7C). In external cohorts, expression of these genes showed independent, stage-dependent associations with progression-free and overall survival.

**Conclusion:**

OMNIS is a spatially informed multi-omics framework that couples accurate prediction with gene-level interpretability. By embedding three-dimensional genome organization into deep-learning models, OMNIS nominates biologically coherent, context-specific drivers of progression and may guide future biomarker development and personalized therapy in precision oncology.

## 1 Background

Cancer continues to be a leading cause of mortality globally, and accurately predicting recurrence and metastasis is crucial for improving patient outcomes and guiding treatment decisions (Siegel et al., 2023). Despite substantial advancements in medical research, predicting cancer recurrence remains challenging. Current methods, including blood tests and imaging, often lack the precision needed for early detection, particularly in distinguishing between primary and metastatic tumors. While adjuvant therapies like targeted treatment and immune checkpoint inhibitors offer promise for early-stage patients at high risk of recurrence, determining the optimal timing and selection of these therapies remain complex. Identifying reliable prognostic factors for early recurrence is therefore essential to help clinicians personalize treatment plans, minimizing overtreatment while effectively addressing metastasis and recurrence risks.

With the rapid development of high-throughput technologies, the application of single-omics data in cancer prognosis and survival prediction has become increasingly widespread. Gene expression data have been shown to have significant value in predicting tumor prognosis (Zaccaria et al., 2024), while changes in deoxyribonucleic acid (DNA) methylation patterns play a key role in the epigenetic regulation of tumors (Peng et al., 2020). However, single-omics data often fail to comprehensively capture the complex biological characteristics of tumors. As a result, the integration of multi-omics data has emerged as an effective strategy to address this challenge. By combining information from different molecular layers, multi-omics approaches provide a more comprehensive biological perspective on cancer, thereby improving the accuracy and robustness of prognosis prediction.

As sequencing costs continue to decline, researchers have increasingly implemented routine multi-omics analyses, integrating DNA sequencing, ribonucleic acid (RNA) sequencing, and methylation analysis to comprehensively characterize the molecular landscape of cancer. This multidimensional approach enhances our understanding of the interrelationships between the genome, transcriptome, and epigenome, significantly deepening insights into the complex biological characteristics of tumors. However, the rapid growth in the volume of multi-omics data is beginning to expose the limitations of traditional analytical methods. Existing frameworks typically reduce high-throughput data to simplified two-dimensional matrices, neglecting critical spatial relationships embedded within the three-dimensional chromatin structure (Franke et al., 2016), which is vital for coordinating genomic interactions and functional accessibility. Overcoming this limitation and integrating complex spatial and functional information is now a central challenge in multi-omics research.

Specifically, three methodological gaps severely constrain the interpretative power of current multi-omics integration approaches. First, linear statistical models fail to capture nonlinear regulatory networks mediated by chromatin topology, such as enhancer-promoter looping that drives synchronized epigenetic modifications and transcriptional bursting (Fulco et al., 2019). Second, stringent multiple-testing corrections (Benjamini and Hochberg, 1995), though necessary for analyzing high-dimensional genomic data with features vastly outnumbering samples, risk dismissing biologically significant spatial co-occurrence events—such as the oncogenic synergy between MYC amplification and hypermethylation of the neighboring miR-34b promoter within the 8q24.21 cancer susceptibility locus. Third, mainstream tools such as Multi-Omics Factor Analysis (MOFA+) (Argelaguet et al., 2020) and iCluster (Shen et al., 2009)—treat molecular features as discrete, linear entities, neglecting their spatial coordination within topologically associating domains (TADs). This oversight hinders the detection of TAD-mediated co-expression patterns or cross-omics regulatory cascades that dependent on native chromatin folding states. Collectively, these limitations underscore the urgent need for unified analytical frameworks that fully respect and leverage the spatial complexity inherent in genomic organization.

Artificial intelligence technologies present novel opportunities to overcome traditional analytical limitations. Over the past decade, deep learning (DL) architectures have driven technological revolutions in domains such as computer vision and speech recognition, yet their medical applications have seen slower adoption, primarily constrained by regulatory demands for transparent interpretation of black-box models. The paradigm-shifting advantages of deep neural networks manifest in two principal dimensions: (1) autonomous identification of intricate nonlinear correlations within data, which may complement conventional feature-wise statistical testing; (2) enhanced robustness to noise through convolutional layer architectures. While opaque models prevail in engineering fields like autonomous driving (Yang et al., 2022), the medical domain’s non-negotiable requirement for decision-making transparency forces researchers to balance model complexity against interpretability. Notably, several artificial intelligence (AI)–based diagnostic tools have already progressed from laboratory validation to enter clinical deployment (Benjamens et al., 2020), marking a critical transition from algorithmic innovation to practical healthcare implementation.

In medical research, model interpretability serves dual imperatives: facilitating the discovery of novel biomarker discovery and elucidating disease pathobiology (Picard et al., 2021). The convergence of artificial intelligence with next-generation sequencing has catalyzed the development of advanced interpretability frameworks, including DeepLIFT (Shrikumar et al., 2017), Integrated Gradients (Sundararajan et al., 2017), and DeepExplain (Samek GM et al., 2019). These methodologies deconstruct the cognitive barriers of conventional “black-box” modeling through quantitative feature attribution analysis (Despraz et al., 2017). In multi-omics integration studies, such breakthroughs enable the synthesis of heterogeneous high-dimensional datasets while preserving biological traceability. The resultant attribution score matrix not only quantifies variable contributions in predictive models but also generates testable biological hypotheses for mechanistic investigations.

Here, we introduce OMics Network Integration and Spatial representation (OMNIS), a novel framework for multi-omics analysis using a convolutional DL model designed to identify hidden, nonlinear patterns in spatially connected, feature-rich, multilayered data. By transforming multi-omics data into a multi-channel spatial genomic feature map, we capture the spatial connections between genes, which are then analyzed through convolutional layers. This approach outperforms traditional non-spatial data transformations. Additionally, the model is enhanced with Integrated Gradients (IG), a non-parametric technique that quantifies the contribution of each feature to the model’s predictions, allowing us to prioritize candidate biomarkers associated with model classification. In this study, we demonstrate that OMNIS can predict tumor recurrence, distinguish between primary and metastatic tumors, and reveal key biological markers related to cancer progression. By integrating various omics data types (mutation, expression, methylation, and copy number) into a single framework, OMNIS enables both accurate classification and detailed interpretation of multi-omics data. Compared with traditional methods, OMNIS provides a more interpretable approach with potential translational relevance for the deep integration of multi-omics data through spatial embedding and convolution-based feature learning. This framework not only enhances prediction accuracy but also offers new opportunities for personalized cancer treatment and targeted therapies.

## 2 Materials and methods

### 2.1 Study overview

**Figure 1:**
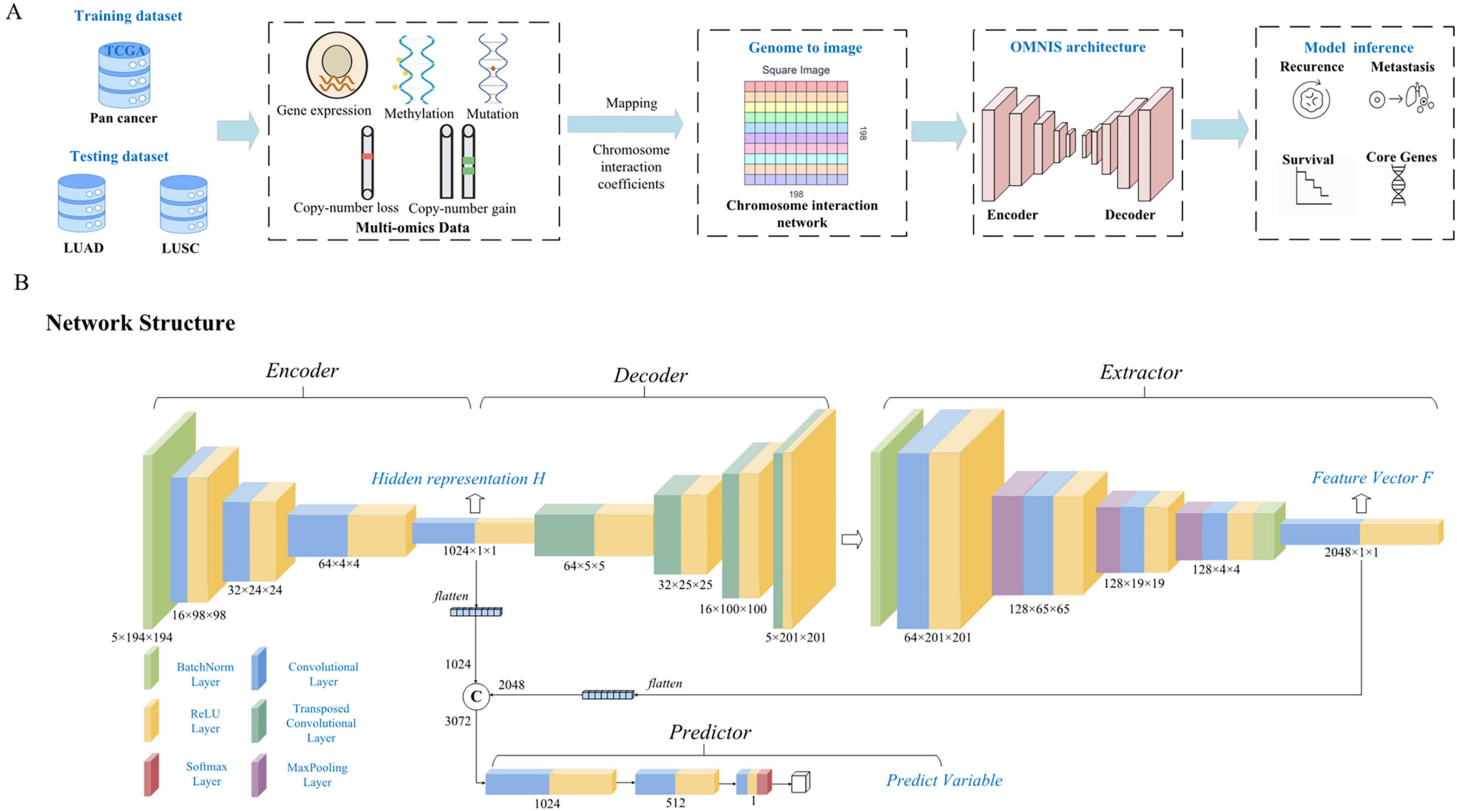
Overall study framework. **(A)** We used the TCGA pan-cancer dataset and mapped multi-omics information into images according to a Hi-C–derived chromatin interaction (chromosome-proximity) network. After model training, we introduced two independent validation cohorts (GSE31210: early-stage lung adenocarcinoma; GSE135222: advanced NSCLC treated with anti-PD-1/PD-L1), representing early- and late-stage disease, to validate the candidate genes identified by the model. The validation pipeline included identifying key genes driving recurrence and metastatic progression, characterizing the hidden representation space of genomic data, and examining its association with survival outcomes. **(B)** Within this model framework, the first part of the network encodes genomic data into a hidden representation H, which is subsequently decoded to reconstruct the corresponding image. The downstream network modules then extract features from the reconstructed image, concatenate them with H, and use the combined representation to perform the final prediction task.

In this study, the OMNIS workflow (Figure 1) comprises four principal modules: (i) multi-omics spatial feature-map construction—somatic mutation, transcriptomic, DNA-methylation, and copy-number features are projected, according to the physical order of genes along the chromosomes, onto genome-wide spatial feature maps in distinct channels, enabling convolutional kernels to capture latent, non-linear spatial dependencies among neighbouring genes; (ii) convolution-based DL classification—these multi-channel images are fed into a deep convolutional network followed by stacked multilayer perceptrons, from which two independent classifiers are derived: one predicts tumor-recurrence risk and the other discriminates primary from metastatic tumors; (iii) integrated-gradients-based interpretability—a non-parametric integrated-gradients approach is applied to each trained classifier to assign attribution scores to individual genes, quantitatively assessing their contributions to the respective predictions; and (iv) Biomarker prioritization and biological annotation—genes are ranked by integrated-gradients scores to nominate candidate biomarkers, which are contextualised through pathway-enrichment analysis to generate testable biological hypotheses related to model predictions. Cross-cohort validation demonstrates that leveraging spatial genomic connectivity markedly enhances the accuracy of both recurrence prediction and primary/metastatic discrimination, while the integrated-gradients–based interpretive framework helps prioritize candidate biomarkers without relying solely on multiple independent statistical tests and provides hypothesis-generating biological insights into tumor progression.

### 2.2 Multi-omics data acquisition and preprocessing

We assembled a pan-cancer compendium of transcriptomic, copy-number, and somatic-mutation profiles together with matched clinical annotations for 33 tumor types in The Cancer Genome Atlas (TCGA). Clinical records were retrieved from the Genomic Data Commons (GDC) portal^1^ and, after excluding (i) normal tissue samples, (ii) cases with missing or “unknown” pathological stage, and (iii) patients lacking any of the four omics layers (copy number, methylation, gene expression or somatic mutation), a total of 1,578 tumors were retained for downstream analyses. Gene-level RNA-seq counts (HTSeq) were obtained from the UCSC Xena GDC Hub and transformed to log₂ (count + 1); counts were further normalized to transcripts-per-million (TPM) to permit inter-sample comparison, following the procedure of the Toil pipeline (Vivian et al., 2017). Gene-centric focal copy-number scores were retrieved from the GISTIC2.0 output (“focal score by gene”) on the same hub; SNP6 copy-number profiles were processed with ASCAT v2.4 (Raine et al., 2016), and segment-level values were normalized for ploidy and purity by dividing each total copy number by the inferred ploidy and subsequently applying a log₂ transformation to yield log-R ratios. Somatic mutation calls were downloaded through TCGAbiolinks for the Muse, MuTect, SomaticSniper and VarScan pipelines and merged by taking the union of gene–position pairs after removal of duplicates, producing a non-redundant catalogue; each missense variant was annotated with PolyPhen-2 to predict structural impact (Adzhubei et al., 2010). Where Infinium HumanMethylation450 data were available, *β*-values were averaged per gene to provide locus-specific methylation metrics.

To independently evaluate the robustness and transferability of our model in non-small cell lung cancer (NSCLC), we further collected two external transcriptomic cohorts from the Gene Expression Omnibus (GEO). The early-stage validation cohort GSE31210 comprises genome-wide gene expression profiles for resected lung adenocarcinomas (pathological stage I–II) with matched clinicopathological annotations and follow-up information, whereas the late-stage validation cohort GSE135222 includes RNA-sequencing data from patients with advanced NSCLC treated with anti-PD-1/PD-L1 immune checkpoint inhibitors and associated clinical response and survival data. For both GSE31210 and GSE135222, gene-level expression matrices and corresponding clinical annotations were downloaded from GEO; probe sets (for microarray data) were mapped to official gene symbols, and when multiple probes mapped to the same gene, their expression values were averaged. Expression values were log₂-transformed and, where necessary, normalized across samples to ensure comparability with the TCGA RNA-seq data. Samples lacking essential outcome information (overall survival, progression-free survival and/or treatment response) or key clinical covariates were excluded. These GEO cohorts were used exclusively as independent validation datasets and were not involved in model training or hyperparameter tuning.

### 2.3 OMNIS algorithm architecture

Multi-omics profiles—gene expression, DNA-methylation, single-nucleotide variation, copy-number deletions and amplifications—were fused into a five-channel spatial genomic feature map, in which each grid position, referred to as a pixel in the computational implementation, encodes one gene for each data layer. Genes were arranged in a single 198 × 198 square according to Hi-C-derived chromosome proximity (4, X, 7, 2, 5, 6, 13, 3, 8, 9, 18, 12, 1, 10, 11, 14, 22, 19, 17, 20, 16, 15, 21), thereby preserving three-dimensional topology. The resulting spatial genomic feature map was passed into a four-stage convolutional network implemented in PyTorch 2.2 and inspired by an auto-encoder but deliberately free of pixel-wise reconstruction loss. (i) An encoder comprising three convolution–max-pool blocks compressed the input to a 128-dimensional hidden representation H, achieving multi-source feature fusion. (ii) A lightweight decoder expanded H back to image space through transposed convolutions; an adversarial discriminator, rather than an ℓ₂ penalty, guided this branch, enabling information-oriented denoising. (iii) The reconstructed image passed through a feature-analysis module containing parallel convolutions with kernel sizes 3, 5 and 7 followed by adaptive pooling, capturing both local motifs and long-range chromosomal interactions. (iv) Extracted features were flattened, concatenated with H and forwarded to two fully connected layers (512 → 256 neurons, ReLU activation, dropout 0.3) that terminated in a two-unit softmax layer, yielding the probability of primary versus metastatic disease.

### 2.4 Model training process

Models were trained on an Nvidia RTX 3090 (24 GB) with the Adagrad optimiser (initial learning rate 9.9 × 10^−5^, weight decay 1 × 10^−6^). The learning rate was halved whenever validation loss plateaued for five consecutive epochs, and early stopping terminated training after ten such plateaux or a maximum of 100 epochs. The batch size was fixed at 256 because the square images fitted comfortably in memory. The adversarial reconstruction branch carried a loss weight of *λ* = 0.1, chosen empirically after preliminary sweeps showed that stronger penalties constrained latent-space flexibility and reduced predictive accuracy. Binary cross-entropy loss drove all classification endpoints, while mean-squared error optimized continuous outcomes. Preserving high-throughput chromosome conformation capture (Hi–C) chromosomal ordering consistently accelerated convergence relative to shuffled controls tested in pilot experiments.

### 2.5 Performance evaluation

To develop a robust and generalizable model, this study applied 10-fold cross-validation to evaluate the performance of each DL and machine learning (ML) model on the validation subset. For each fold, we computed the true positives (TP), true negatives (TN), false positives (FP), and false negatives (FN). Our analysis involved constructing confusion matrices and calculating key performance metrics, including accuracy, sensitivity, specificity, positive predictive value (PPV), negative predictive value (NPV), and the F1 score, which were derived as follows:


$$\text{Accuracy}=\frac{\mathrm{TP}+\mathrm{TN}}{\mathrm{TP}+\mathrm{TN}+\mathrm{FP}+\mathrm{FN}}$$

$$\text{Sensitivity}=\frac{\mathrm{TP}}{\mathrm{TP}+\mathrm{FN}}$$

$$\text{Specificity}=\frac{\mathrm{TN}}{\mathrm{TN}+\mathrm{FP}}$$

$$\mathrm{PPV}=\text{Precision}=\frac{\mathrm{TP}}{\mathrm{TP}+\mathrm{FP}}$$

$$\mathrm{NPV}=\frac{\mathrm{TN}}{\mathrm{TN}+\mathrm{FN}}$$

$$F1\;\text{score}=\frac{2*\text{Precision}*\text{Recall}}{\text{Precision}+\text{Recall}}$$

Additionally, we estimated the 95% confidence intervals (CI) for the cross-validated area under the receiver operating characteristic curve (AUROC) and area under the precision–recall curve (AUPR) of each predictive model using bootstrapping with 1,000 iterations.

All ablation metrics across recurrence and primary-versus-metastatic classification tasks are summarized in Supplementary Table S1. Head-to-head benchmark comparisons between OMNIS and classical machine learning methods, including XGBoost and SVM, as well as existing multi-omics integration approaches, including DeepProg and MOFA+, were conducted using the same cohort and evaluation criteria. Comprehensive benchmark metrics for the two classification endpoints are summarized in Supplementary Table S2. Full standardized settings for gene-grid mapping, Hi–C chromosomal ordering, network architecture, hyperparameters, dataset partitioning, and evaluation pipelines are provided in Supplementary Table S3. Together, these supplementary analyses support the robustness of OMNIS and clarify the methodological basis of the reported results.

### 2.6 Model interpretability analysis via integrated gradients

Upon completion of training the model with data, the relevant loss function, output layer, and model weights were stored in a “.pb” file. The Integrated Gradients (IG) method (Sundararajan et al., 2017) was implemented using Captum, a PyTorch-based model interpretability library, to evaluate the trained model and compute feature attribution scores. IG serves as an attribution method that allocates an “attribution score” to each feature within the input data, grounded in the model’s predictions. This score is obtained by computing the gradient of each feature in the image channels relative to the model’s output. Such information indicates which features most strongly support or oppose a given prediction. The attribution maps produced are then employed to extract data from each omics channel and gene-associated grid position. Given that pixels correspond to individual genes, this data can be reformatted and filtered to emphasize the most crucial genes from each data source in the analysis. To accommodate the biological variances across different cancer types, all attribution scores were rescaled using a min-max scaling technique.

## 3 Results

**Figure 2:**
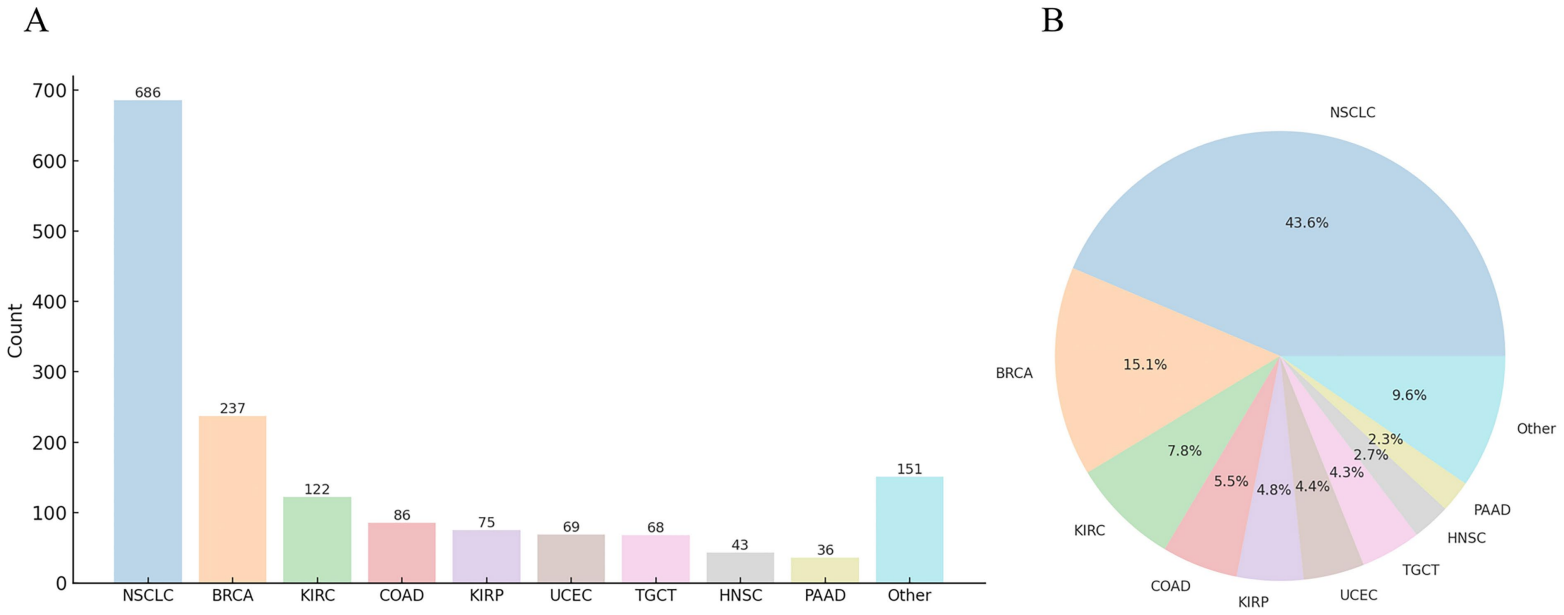
Distribution of tumor types in the study cohort (*n* = 1,573). **(A)** Bar plot showing the number of samples for each tumor type. **(B)** Corresponding pie chart displaying the relative proportions of each tumor type in the cohort. Tumor types accounting for less than 2% of all cases were grouped into the “Other” category, which includes THYM (thymoma), LIHC (liver hepatocellular carcinoma), BLCA (bladder urothelial carcinoma), READ (rectum adenocarcinoma), MESO (mesothelioma), ESCA (esophageal carcinoma), STAD (stomach adenocarcinoma), CHOL (cholangiocarcinoma), OV (ovarian cancer), CESC (cervical squamous cell carcinoma and endocervical adenocarcinoma), and THCA (thyroid carcinoma).

As illustrated in Figures 2A,B, a total of 1,578 tumors from TCGA were retained in our study, including 1,470 primary (93.2%) and 108 metastatic (6.8%) cases. Among the 1,573 tumors with complete histological annotation shown in the figure, NSCLC represented the largest subgroup (686/1,573, 43.6%), followed by breast invasive carcinoma (BRCA; 237, 15.1%), kidney renal clear cell carcinoma (KIRC; 122, 7.8%), colon adenocarcinoma (COAD; 86, 5.5%), kidney renal papillary cell carcinoma (KIRP; 75, 4.8%), uterine corpus endometrial carcinoma (UCEC; 69, 4.4%), testicular germ cell tumors (TGCT; 68, 4.3%), head and neck squamous cell carcinoma (HNSC; 43, 2.7%), and pancreatic adenocarcinoma (PAAD; 36, 2.3%). Less prevalent tumor types were pooled into an “Other” category (151, 9.6%), comprising THYM (thymoma), LIHC (liver hepatocellular carcinoma), BLCA (bladder urothelial carcinoma), READ (rectum adenocarcinoma), MESO (mesothelioma), ESCA (esophageal carcinoma), STAD (stomach adenocarcinoma), CHOL (cholangiocarcinoma), OV (ovarian cancer), CESC (cervical squamous cell carcinoma and endocervical adenocarcinoma), and THCA (thyroid carcinoma). Recurrence information was available for all patients, with 1,153/1,578 (73.1%) showing no documented recurrence during follow-up and 425/1,578 (26.9%) experiencing tumor relapse.

To further validate our findings across disease stages and treatment contexts, we analyzed two independent NSCLC cohorts. First, the GSE31210 dataset of resected lung adenocarcinomas provided an early-stage validation cohort of 226 patients with stage I–II disease after harmonization of clinical annotations. The median age at diagnosis was 61 years (range, 30–76 years), with a nearly balanced sex distribution (53.5% female) and smoking status (50.9% never-smokers vs. 49.1% ever-smokers). During follow-up, 64 patients (28.3%) developed tumor recurrence or progression, and 35 (15.5%) died, whereas 162 (71.7%) remained recurrence-free and 191 (84.5%) were alive at last contact. Second, to assess robustness in advanced disease under immunotherapy, we included GSE135222, comprising 27 patients with advanced NSCLC treated with immune checkpoint inhibitors. This cohort was predominantly male (81.5%), with a median age of 62 years (range, 38–81 years); 21/27 patients (77.8%) experienced disease progression, while 6/27 (22.2%) had no documented progression at the time of censoring.

**Figure 3:**
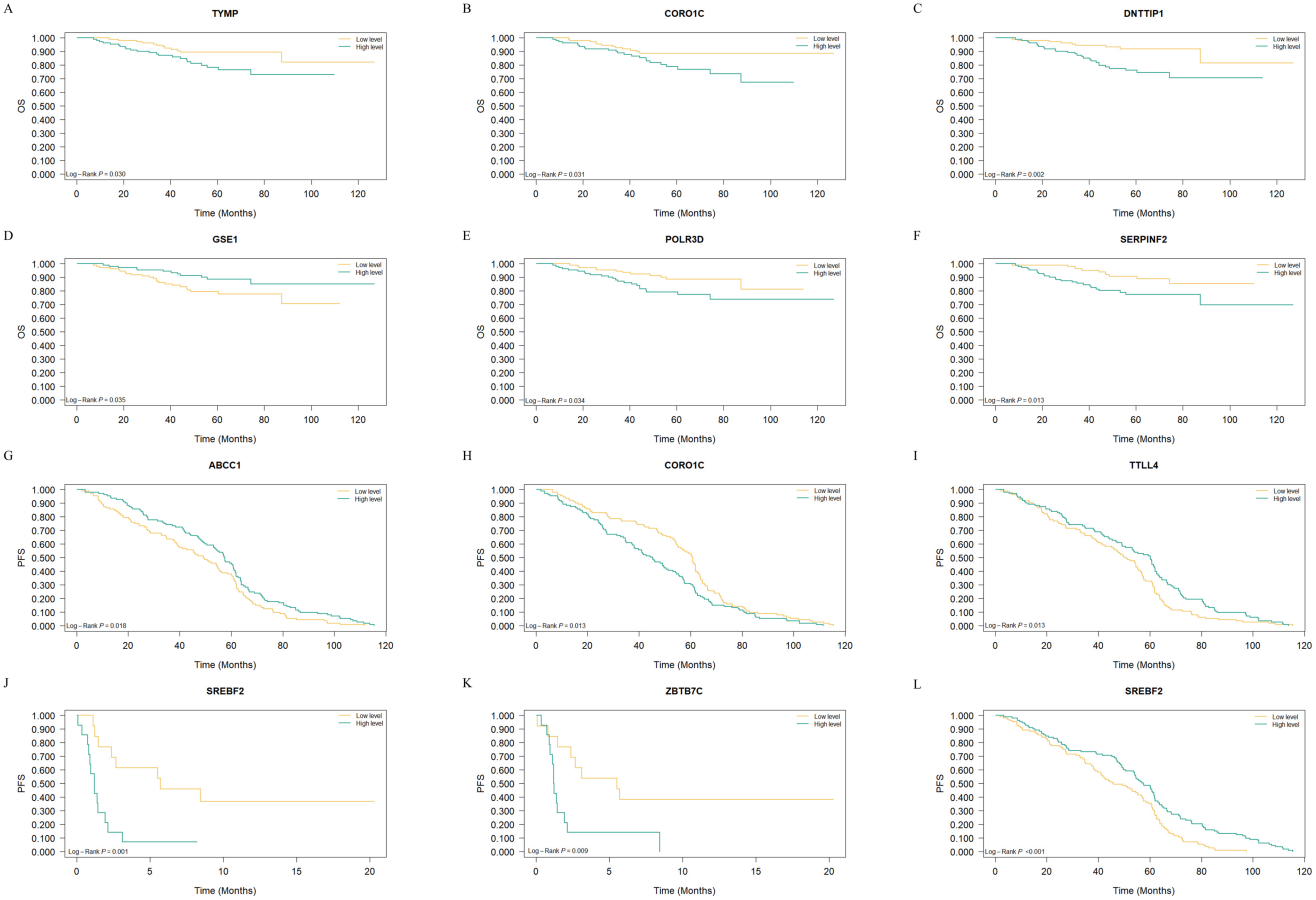
Kaplan–Meier survival analysis of representative model-selected genes in NSCLC cohorts. Kaplan–Meier curves show OS **(A–F)** and PFS **(G–L)** for high vs. low gene expression (median split). Panels A–I and K are from GSE31210 (~120-month follow-up); panels J and L are from GSE135222 (~20-month follow-up). *p* values were calculated by the log-rank test; tick marks indicate censoring.

We constructed a multi-omics–based prediction model to estimate the risk of recurrence and metastasis, and evaluated its performance in both the training and testing cohorts using AUROC, AUPR, and threshold-based classification metrics (Table 1). For recurrence, the model showed excellent discrimination, with an AUROC of 0.956 (95% CI 0.946–0.966) and an AUPR of 0.897 (95% CI 0.873–0.919) in the training set, and an AUROC of 0.970 (95% CI 0.956–0.983) and an AUPR of 0.937 (95% CI 0.904–0.963) in the testing set. Accuracy was comparable between the two cohorts (0.873 vs. 0.882), with consistently high sensitivity (0.929 and 0.932) and specificity (0.852 and 0.862). The model yielded a high negative predictive value (NPV, 0.970 in both sets), whereas the positive predictive value (PPV, 0.698 and 0.724) and F1 scores (0.797 and 0.815) indicated balanced performance in correctly identifying patients with recurrence. For metastasis, the model also performed strongly, with AUROCs of 0.976 (95% CI 0.962–0.986) and 0.980 (95% CI 0.951–0.997), and AUPRs of 0.849 (95% CI 0.786–0.902) and 0.883 (95% CI 0.779–0.963) in the training and testing sets, respectively. Accuracy increased from 0.888 to 0.911, accompanied by very high sensitivities (0.926 and 0.968) and specificities (0.885 and 0.907). As expected in a likely class-imbalanced setting, NPVs were close to 1.0 (0.994 and 0.998), while PPVs remained modest (0.372 and 0.423), resulting in F1 scores of 0.531 and 0.588. Overall, these results suggest that the model showed stable performance between the training and testing cohorts and may be useful for risk stratification, particularly for identifying samples with a lower predicted probability of recurrence or metastatic progression. Beyond global prediction performance, we next examined the prognostic relevance of individual genes highlighted by the model. Within the pan-cancer recurrence model, OMNIS prioritized seven recurrence-associated genes with consistently high attribution scores (IBA57, DNTTIP1, CORO1C, SLC20A2, ABHD17C, TMEM201 and GSE1), and ten metastasis-associated genes in the metastasis model (DNTTIP1, SERPINF2, ABCC1, PLXNA1, BSG, TYMP, POLR3D, TTLL4, SREBF2 and ZBTB7C). To assess whether these model-derived genes were associated with patient outcome, we performed multivariable Cox regression and Kaplan–Meier analyses stratified by gene expression (Tables 2, 3; Figure 3). Among recurrence-associated genes, higher IBA57 expression in the early-stage cohort was associated with improved overall survival (OS; HR = 0.662, 95% CI 0.478–0.918; *p* = 0.013), whereas higher DNTTIP1 expression was associated with worse OS (HR = 1.429, 95% CI 1.113–1.833; *p* = 0.005) and shorter progression-free survival (PFS; HR = 1.211, 95% CI 1.063–1.379; *p* = 0.004). In the late-stage cohort, IBA57, SLC20A2, ABHD17C and TMEM201 were all associated with an increased risk of progression, with HRs ranging from 1.578 to 1.879 and *p* values between 0.024 and 0.045. Kaplan–Meier curves for representative recurrence-associated genes showed clear separation of OS and PFS between high- and low-expression groups, in line with the Cox estimates (Figure 3).

**Table 1:** Predictive performance of the OMNIS model for recurrence and metastasis in the training and testing cohorts.

Outcome	AUROC (95% CI)	AUPR (95% CI)	Accuracy	Sensitivity	Specificity	PPV	NPV	F1 score
Recurrence								
Training set	0.956 (0.946–0.966)	0.897 (0.873–0.919)	0.873	0.929	0.852	0.698	0.970	0.797
Testing set	0.970 (0.956–0.983)	0.937 (0.904–0.963)	0.882	0.932	0.862	0.724	0.970	0.815
Metastasis								
Training set	0.976 (0.962–0.986)	0.849 (0.786–0.902)	0.888	0.926	0.885	0.372	0.994	0.531
Testing set	0.980 (0.951–0.997)	0.883 (0.779–0.963)	0.911	0.968	0.907	0.423	0.998	0.588

AUROC, area under the receiver operating characteristic curve; AUPR, area under the precision–recall curve; PPV, positive predictive value; NPV, negative predictive value.

**Table 2:** Multivariable Cox regression analysis of recurrence-associated genes in early- and late-stage cohorts.

Gene	Stage	Outcome	HR (95% CI)	*p*-value
IBA57	Early-stage	OS	0.662 (0.478–0.918)	**0.013**
DNTTIP1	Early-stage	OS	1.429 (1.113–1.833)	**0.005**
DNTTIP1	Early-stage	PFS	1.211 (1.063–1.379)	**0.004**
IBA57	Late stage	PFS	1.609 (1.010–2.562)	**0.045**
SLC20A2	Late stage	PFS	1.578 (1.026–2.429)	**0.038**
ABHD17C	Late stage	PFS	1.879 (1.085–3.254)	**0.024**
TMEM201	Late stage	PFS	1.854 (1.035–3.321)	**0.038**

HR, hazard ratio; CI, confidence interval; OS, overall survival; PFS, progression-free survival.

Hazard ratios were estimated using multivariable Cox proportional hazards models adjusted for age, sex, and smoking status.

Bold *p*-values indicate statistical significance (*p* < 0.05).

**Table 3:** Multivariable Cox regression analysis of metastasis-associated genes in early- and late-stage cohorts.

Gene	Stage	Outcome	HR (95% CI)	*p*-value
PLXNA1	Early-stage	PFS	1.158 (1.001–1.339)	**0.049**
POLR3D	Early-stage	PFS	1.167 (1.030–1.322)	**0.016**
TTLL4	Early-stage	PFS	0.844 (0.738–0.965)	**0.013**
SREBF2	Early-stage	PFS	0.849 (0.742–0.970)	**0.016**
TYMP	Early-stage	PFS	1.160 (1.009–1.333)	**0.038**
SREBF2	Late stage	OS	2.018 (1.235–3.297)	**0.005**
ZBTB7C	Late stage	OS	1.670 (1.094–2.549)	**0.018**

HR, hazard ratio; CI, confidence interval; OS, overall survival; PFS, progression-free survival.

Hazard ratios were estimated using multivariable Cox proportional hazards models adjusted for age, sex, and smoking status.

Bold *p*-values indicate statistical significance (*p* < 0.05).

Among metastasis-associated genes, Cox models revealed distinct stage-specific patterns (Table 3). In the early-stage cohort, higher PLXNA1, POLR3D and TYMP expression was associated with an increased risk of progression (HR = 1.158, 95% CI 1.001–1.339; *p* = 0.049; HR = 1.167, 95% CI 1.030–1.322; *p* = 0.016; HR = 1.160, 95% CI 1.009–1.333; *p* = 0.038, respectively), whereas higher TTLL4 and SREBF2 expression was associated with a reduced risk of progression (HR = 0.844, 95% CI 0.738–0.965; *p* = 0.013; HR = 0.849, 95% CI 0.742–0.970; *p* = 0.016). In the late-stage cohort, SREBF2 and ZBTB7C were associated with shorter OS (HR = 2.018, 95% CI 1.235–3.297; *p* = 0.005; HR = 1.670, 95% CI 1.094–2.549; *p* = 0.018). Consistently, Kaplan–Meier curves for these metastasis-associated genes showed divergent survival trajectories between expression-defined subgroups (Figure 3).

## 4 Discussion

In this study, OMNIS prioritized a compact set of seven recurrence-associated genes (IBA57, DNTTIP1, CORO1C, SLC20A2, ABHD17C, TMEM201 and GSE1) and ten metastasis-associated genes (DNTTIP1, SERPINF2, ABCC1, PLXNA1, BSG, TYMP, POLR3D, TTLL4, SREBF2 and ZBTB7C) at the pan-cancer level. These model-derived candidates repeatedly ranked among the top features across multiple cancer types and showed consistent alterations between event and non-event groups at the multi-omics levels. Importantly, Kaplan–Meier and multivariable Cox analyses confirmed that a subset of these genes carries independent prognostic information: IBA57, DNTTIP1, SLC20A2, ABHD17C and TMEM201 were associated with recurrence risk, whereas PLXNA1, POLR3D, TTLL4, SREBF2, TYMP and ZBTB7C were associated with progression or overall survival in stage-stratified cohorts. Several genes exhibited stage- or endpoint-dependent associations—for example, IBA57 was protective for overall survival in early-stage disease but associated with shorter progression-free survival in advanced-stage tumors, and SREBF2 switched from a favorable PFS marker in early-stage patients to an adverse OS marker in late-stage metastatic disease. Exploratory analyses also revealed discordant behavior between different endpoints for some genes such as SERPINF2. Rather than representing contradictions, these patterns likely reflect the dynamic interplay between treatment exposure, clonal evolution and competing risks along the disease course, and highlight that the prognostic impact of individual genes may be context- and stage-specific.

We developed OMNIS as a pan-cancer, multi-omics framework that systematically interrogates the molecular determinants of recurrence and distant metastasis across cancer types. In OMNIS, multi-omics data are mapped into 198 × 198 spatial genomic feature maps and genes are reordered according to 3-D chromosomal contacts, integrating somatic mutations, copy-number alterations, transcriptomic profiles and epigenetic features into a unified spatial representation. This configuration substantially improved the accuracy of the deep-learning model in predicting tumor recurrence and enabled precise discrimination between primary and metastatic tumors. The entire pipeline operates on raw multi-omics matrices with virtually no additional preprocessing, markedly lowering the barrier to reproducibility. This spatial layout retains cis- and trans-regulatory cues and provides Hi–C–like topological priors, allowing convolutional kernels to capture inter-chromosomal and hierarchical interaction features. This design is further supported by recent Hi–C-based computational studies. Sefer showed that Hi–C interaction graphs can capture the contribution of histone-modification pairs to chromatin shape, indicating that epigenetic marks are closely associated with Hi–C interactions and TAD organization (Sefer, 2021). ProbC further demonstrated that Hi–C/Micro–C contacts can be jointly modeled with epigenomic and transcription-related features, supporting the concept that three-dimensional chromatin interactions are coupled with multiple functional genomic layers (Sefer, 2022). In addition, deconvolution analysis of ensemble 3C/Hi–C matrices revealed latent subpopulation-specific chromatin contact structures, suggesting that chromatin topology may encode biologically meaningful heterogeneity rather than serving merely as a technical spatial constraint (Sefer et al., 2016). Together, these studies reinforce the rationale of OMNIS, in which multi-omics features are embedded into a spatially organized genomic representation to enable convolutional filters to learn both local and long-range regulatory dependencies informed by three-dimensional genome organization. Compared with deep-learning frameworks such as DeepProg, DeepKEGG and meta-learning–based survival models, which typically rely on pre-aggregated pathway scores or hand-crafted feature engineering, OMNIS directly learns from genome-wide multi-omics tensors while preserving spatial context, thereby reducing information loss at the feature-construction step and enabling downstream interpretability at single-gene resolution. Within the OMNIS framework, a reconstruction-free autoencoder automatically selects the genomic image representation most suitable for the prediction task and generates a 128-dimensional latent code, preserving the input distribution without incurring reconstruction loss. Coupled with integrated gradients, OMNIS additionally produces per-gene attribution scores that allow rapid prioritization of pathways and loci associated with recurrence and metastasis predictions through non-parametric, readily interpretable importance values—opening the “black box” of convolutional neural network decision-making and linking model performance to testable molecular hypotheses. Consistent with this, the OMNIS prediction models achieved excellent discrimination in both training and testing cohorts, with AUROCs of 0.956–0.970 and AUPRs of 0.897–0.937 for recurrence, and AUROCs of 0.976–0.980 and AUPRs of 0.849–0.883 for metastasis, while maintaining high accuracy, sensitivity, specificity and NPVs, supporting its potential utility for recurrence/metastasis risk stratification in retrospective multi-omics cohorts. At the biological level, the recurrence-associated gene set converges on mitochondrial metabolism, chromatin regulation and structural remodeling. IBA57 participates in mitochondrial iron–sulfur cluster assembly and oxidative phosphorylation and has been shown to be essential for cancer cell survival under acidic conditions (Michl et al., 2022). In our data, higher IBA57 expression was associated with improved overall survival in early-stage disease, suggesting that preserved mitochondrial function may mark less aggressive biology at diagnosis, but the same pathway was associated with shorter PFS in advanced-stage tumors, consistent with a role in sustaining residual disease under therapeutic stress. DNTTIP1 is a chromatin-associated factor that recruits histone deacetylases and activates MAPK signalling, and has been reported to drive metastasis and act as an independent predictor of poor prognosis in nasopharyngeal carcinoma (Ding et al., 2022). In line with these findings, higher DNTTIP1 expression in our cohort was associated with worse OS and PFS in early-stage patients, and DNTTIP1 was independently highlighted in both recurrence and metastasis models, suggesting that it may represent a candidate epigenetic biomarker associated with relapse and dissemination. SLC20A2, a sodium-dependent phosphate cotransporter from the SLC20 family, is closely linked to tumor growth rates and phosphate handling in cancer (Kang et al., 2020); ABHD17C, a depalmitoylase stabilized by USP35, exerts oncogenic effects through activation of PI3K/AKT signalling in hepatocellular carcinoma (Wang et al., 2023); and TMEM201, a nuclear-envelope protein, has been associated with hepatocellular carcinoma progression and shorter survival (Chen et al., 2023). In our recurrence models, higher SLC20A2, ABHD17C and TMEM201 expression correlated with increased risk of progression in advanced-stage disease, suggesting that OMNIS may prioritize candidate features associated with phosphate and lipid metabolism, membrane and nuclear architecture, and stress-tolerant growth. CORO1C and GSE1, which contribute to cytoskeletal remodeling, migration and chromatin organization, were also consistently prioritized, aligning with a model in which local relapse depends on both metabolic resilience and structural plasticity of tumor cells under therapy.

The metastasis-associated gene set identified by OMNIS maps onto several hallmarks of metastatic competence, including enhanced motility, microenvironmental remodeling and metabolic reprogramming. PLXNA1, a plexin family receptor that functions as a neuropilin co-receptor in axon-guidance and cell-migration pathways, is elevated in multiple solid tumors and has been linked to tumor progression, metastasis and therapy resistance (Hu et al., 2024). Here, higher PLXNA1 expression was associated with increased risk of progression in early-stage patients, suggesting a role in early dissemination or micrometastatic outgrowth. POLR3D encodes a subunit of RNA polymerase III involved in cell-cycle control and biosynthetic activity (Beuzelin and Kaeffer, 2018); its adverse association with PFS is consistent with enhanced transcriptional output in aggressively proliferating clones. In contrast, TTLL4, which modulates exosome biogenesis and *β*-tubulin polyglutamylation and has been implicated in brain metastasis (Arnold et al., 2020), showed a protective association with PFS in early-stage disease, hinting at context-dependent effects of microtubule and vesicle-trafficking states during the metastatic cascade. SREBF2, a key transcriptional regulator of cholesterol metabolism and scavenger receptor expression (Wang et al., 2024), exemplified a stage-dependent pattern: higher expression correlated with longer PFS in early-stage disease but with shorter OS in advanced-stage metastatic tumors, consistent with a shift from potentially favourable metabolic states early on to cholesterol-dependent, therapy-resistant phenotypes later in the disease course. TYMP, a nucleotide-metabolism enzyme and potential prognostic marker in glioma (Zhang et al., 2024), showed an adverse association with PFS in early-stage patients, in line with the concept that reprogrammed nucleotide metabolism supports proliferative and invasive behavior. ZBTB7C, a POZ/BTB and zinc-finger transcription factor that can interact with p53 (Jeon et al., 2012), emerged as an adverse OS marker in advanced-stage metastasis, consistent with its reported involvement in transcriptional regulation in renal carcinoma. Other OMNIS-prioritized genes in the metastasis set, such as ABCC1 and BSG—encoding a drug-efflux pump and a matrix-remodeling receptor, respectively—fit well with established roles in chemoresistance and invasive behavior, while SERPINF2, involved in coagulation and fibrinolysis, links vascular and matrix dynamics to metastatic colonization.

Together, these findings indicate that OMNIS may identify candidate molecular patterns associated with recurrence and distant metastasis across tissues of origin, rather than solely reflecting cancer type–specific signals. At the same time, the stage- and endpoint-dependent associations observed for genes such as IBA57, SREBF2 and SERPINF2 underscore the complexity of translating multi-omics signatures into static “good” or “bad” markers. Prognostic effects may vary across disease stages and therapeutic contexts, and may differ between early progression and long-term survival endpoints, reflecting changes in treatment exposure, clonal competition and host responses. In this sense, OMNIS should be viewed as a hypothesis-generating tool: it nominates a biologically coherent but nuanced set of candidate biomarkers whose roles in the recurrence–metastasis continuum warrant dedicated longitudinal and mechanistic studies.

Although OMNIS delivers outstanding performance without onerous preprocessing, several avenues for refinement remain. First, while the current 198 × 198 genomic-projection layout provided the best results in our benchmarks, incorporating topologically associating domains or higher-resolution Hi–C contact maps could further capture subtler cis- and trans-regulatory signals. Second, the framework is highly amenable to modular expansion: external pathology images, more granular somatic-mutation annotations and metabolomic data could be introduced as additional channels, enabling cross-modal co-learning through a shared latent space and allowing OMNIS to be compared head-to-head with image-centric or mutation-centric deep models. Third, validation in longitudinal cohorts and multi-centre datasets is warranted to assess the network’s generalizability across sequencing platforms, populations and therapeutic contexts, and to experimentally confirm the functional roles of integrated-gradient–highlighted genes *in vitro* and *in vivo*. Despite these limitations, our results demonstrate that embedding 3-D genomic organization into deep multi-omics models provides a useful research strategy not only for predicting recurrence and distant metastasis, but also for nominating a focused and biologically rich set of hypothesis-generating candidate biomarkers associated with metabolic rewiring, chromatin remodeling and cytoskeletal dynamics to clinically manifest disease progression.

## 5 Conclusion

This work proposes and validates a spatially informed multi-omics DL strategy: genes are mapped into a 198 × 198 image according to chromosomal contacts, then processed with a convolutional neural network and a reconstruction-free autoencoder to deliver high-accuracy predictions of tumor recurrence and robust separation of primary and metastatic lesions. The approach offers three key advantages: (i) explicit incorporation of 3-D genome structure markedly boosts model performance; (ii) the autoencoder–IG combination yields a concise, interpretable hidden representation while providing per-gene importance scores that facilitate candidate biomarker prioritization and hypothesis generation; and (iii) the same framework can flexibly accommodate additional channels to integrate epigenomic, Hi–C and other data layers for more complex multi-omics scenarios. Future work will extend OMNIS to larger, multicenter and prospective cohorts and systematically investigate how channel composition and network architecture influence model generalizability, thereby advancing the integration of 3-D genomics and DL in translational cancer research.

## Data Availability

The original contributions presented in the study are included in the article/Supplementary material, further inquiries can be directed to the corresponding author.

## Footnotes

1
https://portal.gdc.cancer.gov/projects

## Glossary

AUPR: Area under the precision–recall curve
AUROC: Area under the receiver operating characteristic curve
AI: Artificial intelligence
ASCAT: Allele-specific copy number analysis of tumors
BLCA: Bladder urothelial carcinoma
BRCA: Breast invasive carcinoma
CESC: Cervical squamous cell carcinoma and endocervical adenocarcinoma
CHOL: Cholangiocarcinoma
CI: Confidence interval
COAD: Colon adenocarcinoma
DL: Deep learning
DNA: Deoxyribonucleic acid
ESCA: Esophageal carcinoma
FP: False positive
FN: False negative
GDC: Genomic data commons
GEO: Gene expression omnibus
GISTIC2.0: Genomic identification of significant targets in cancer 2.0
Hi-C: High-throughput chromosome conformation capture
HNSC: Head and neck squamous cell carcinoma
HR: Hazard ratio
HTSeq: High-throughput sequencing framework
KIRC: Kidney renal clear cell carcinoma
KIRP: Kidney renal papillary cell carcinoma
LIHC: Liver hepatocellular carcinoma
MAPK: Mitogen-activated protein kinase
MESO: Mesothelioma
MOFA+: Multi-omics factor analysis
ML: Machine learning
NPV: Negative predictive value
NSCLC: Non-small cell lung cancer
OS: Overall survival
OV: Ovarian cancer
PAAD: Pancreatic adenocarcinoma
PD-1: Programmed cell death protein 1
PD-L1: Programmed cell death ligand 1
PFS: Progression-free survival
PI3K: Phosphoinositide 3-kinase
PPV: Positive predictive value
READ: Rectum adenocarcinoma
ReLU: Rectified linear unit
RNA: Ribonucleic acid
SNP6: SNP array 6.0
STAD: Stomach adenocarcinoma
TCGA: The cancer genome atlas
TGCT: Testicular germ cell tumors
THCA: Thyroid carcinoma
THYM: Thymoma
TN: True negative
TP: True positive
TPM: Transcripts-per-million
UCEC: Uterine corpus endometrial carcinoma
UCSC: University of California, Santa Cruz
OMNIS: OMics network integration and spatial representation
IG: Integrated gradients
