# Supplementary material for: OMNIS: a spatially informed multi-omics deep-learning framework for tumor recurrence prediction and primary–metastatic tumor differentiation title page
Source: Front Artif Intell. 2026 Jul 15;9:1817043. doi: 10.3389/frai.2026.1817043 (PMC13416578; doi:10.3389/frai.2026.1817043)
Supplement: Supplementary file 1 [file Data_Sheet_1.PDF]

Supplementary Table 1. Control and ablation analyses evaluating the contribution of Hi-C-informed spatial embedding in OMNIS

Analysis purpose	Model / control setting	Spatial organization	Omics input	AUROC/ AUPR recurrence	AUROC/AUPR primary–metastatic	Interpretation
Full model	OMNIS	Hi-C-informed ordering	Full multi-omics	0.970/0.937	0.980/0.883	Reference model
Test contribution of spatial layout	w/o Hi-C-guided ordering	Shuffled or non-spatial ordering	Full multi-omics	0.895/0.835	0.874/0.698	Performance decreased after disrupting spatial gene adjacency
Test contribution of multi-omics integration	Reduced omics input	Hi-C-informed ordering	Reduced omics channels	0.895/0.784	0.851/0.642	Performance decreased when omics layers were reduced
Test contribution of feature-fusion module	w/o autoencoder fusion	Hi-C-informed ordering	Full multi-omics	0.881/0.812	0.865/0.672	Performance decreased after removing latent feature fusion
Test non-spatial multi-omics integration	MOFA+	No spatial ordering	Full multi-omics latent factors	0.768/0.601	0.794/0.418	Non-spatial latent-factor integration showed lower performance
Test non-spatial deep-learning representation	DeepProg	No spatial ordering	Multi-omics features	0.843/0.712	0.861/0.582	Non-spatial deep-learning baseline showed lower performance
Test cancer-type transferability	Leave-one-cancer-type-out OMNIS	Hi-C-informed ordering	Full multi-omics	0.912/0.848	0.924/0.756	Evaluates whether performance persists when cancer types are held out

Supplementary Table 2. Benchmark comparison of OMNIS with conventional machine-learning and multi-omics integration methods

Method	AUROC (95%CI)	AUPR (95%CI)	Accuracy	Sensitivity	Specificity	PPV	NPV	F1
**Recurrence**								
OMNIS	0.970 (0.956–0.983)	0.937 (0.904–0.963)	0.882	0.932	0.862	0.724	0.970	0.815
DeepProg	0.843 (0.812–0.874)	0.712 (0.658–0.764)	0.794	0.801	0.791	0.582	0.901	0.674
MOFA+	0.768 (0.731–0.804)	0.601 (0.542–0.658)	0.742	0.725	0.751	0.498	0.885	0.591
XGBoost	0.720 (0.682–0.758)	0.550 (0.501–0.601)	0.685	0.650	0.700	0.482	0.824	0.554
SVM	0.680 (0.641–0.721)	0.480 (0.431–0.531)	0.629	0.580	0.650	0.415	0.783	0.484
**Primary–metastatic classification**								
OMNIS	0.980 (0.951–0.997)	0.883 (0.779–0.963)	0.911	0.968	0.907	0.423	0.998	0.588
DeepProg	0.861 (0.824–0.896)	0.582 (0.498–0.664)	0.845	0.724	0.854	0.198	0.982	0.311
MOFA+	0.794 (0.751–0.835)	0.418 (0.338–0.502)	0.798	0.652	0.809	0.142	0.974	0.233
XGBoost	0.750 (0.710–0.790)	0.320 (0.258–0.382)	0.731	0.480	0.750	0.126	0.950	0.200
SVM	0.700 (0.658–0.742)	0.240 (0.185–0.295)	0.696	0.380	0.720	0.093	0.939	0.149

Supplementary Table 3. Implementation details of OMNIS architecture, data mapping, and evaluation procedure

**Reviewer concern**	**Clarified implementation detail**	**Setting used in OMNIS**
Gene-to-pixel mapping	Genes were first arranged according to the Hi-C-derived chromosome order and then sorted by genomic coordinates within each chromosome. The ordered gene list was filled into the 2D matrix in a row-wise manner.	One valid grid position corresponds to one gene; the same gene-to-grid index was used across all omics channels.
Determination of 198 × 198 size	The side length was selected as the smallest square grid sufficient to accommodate the final retained gene set after preprocessing.	198 × 198 grid, corresponding to 39,204 available grid positions.
Handling of unused grid positions	Grid positions not assigned to any gene were treated as padding.	Padding positions were set to zero across all channels and excluded from gene-level attribution and biomarker interpretation.
Input omics channels	Multi-omics features were organized into a multi-channel spatial genomic feature map.	Gene expression, DNA methylation, single-nucleotide variation, copy-number deletion, and copy-number amplification.
Source of Hi-C-derived ordering	Chromosome ordering was based on normalized inter-chromosomal Hi-C contact frequencies. Chromosomes with stronger spatial proximity were placed closer in the spatial genomic feature map.	Chromosome order: 4, X, 7, 2, 5, 6, 13, 3, 8, 9, 18, 12, 1, 10, 11, 14, 22, 19, 17, 20, 16, 15, 21.
Encoder architecture	The encoder compressed the multi-channel input into a latent representation using convolution and pooling.	Three convolution–max-pooling blocks; 128-dimensional hidden representation.
Decoder branch and "reconstruction-free" wording	The decoder branch was included, but it was not optimized using conventional pixel-wise reconstruction loss.	The wording was revised to indicate that the model is free of pixel-wise reconstruction loss rather than free of a decoder.
Decoder regularization	The decoder branch was guided by adversarial regularization.	Adversarial decoder branch weight $\lambda = 0.1$.
Classification module	Features from the convolutional feature-analysis module and latent representation were used for classification.	Fully connected layers: 512 → 256 neurons, ReLU activation, dropout = 0.3, task-specific binary output layer.

Classification loss	Classification endpoints were optimized using binary cross-entropy.	Binary cross-entropy loss for recurrence prediction and primary–metastatic classification.
Pixel-wise reconstruction loss	No conventional reconstruction loss was used.	No L1, L2, or mean-squared pixel-wise reconstruction loss was applied to decoder output.
Training/testing split	The TCGA cohort was divided into training and held-out testing sets using stratified sampling.	Stratification considered clinical outcome and cancer type whenever feasible.
Cross-validation and hyperparameter tuning	Model development and hyperparameter selection were performed within the training set.	10-fold cross-validation; held-out testing set was not used for model selection or hyperparameter tuning.
External validation datasets	External GEO cohorts were not used for model training or hyperparameter tuning.	GSE31210 and GSE135222 were used for independent gene-level prognostic follow-up.
